# Wealth status and sex differential of household head: implication for source of drinking water in Nigeria

**DOI:** 10.1186/s13690-015-0105-9

**Published:** 2015-11-25

**Authors:** Oyewale Mayowa Morakinyo, Stephen Ayo Adebowale, Elizabeth Omoladun Oloruntoba

**Affiliations:** Department of Environmental Health Sciences, Faculty of Public Health, College of Medicine, University of Ibadan, Ibadan, Nigeria; Department of Epidemiology and Medical Statistics, Faculty of Public Health, College of Medicine, University of Ibadan, Ibadan, Nigeria

**Keywords:** Potable water, Household head, Wealth index, Nigeria

## Abstract

**Background:**

Source of potable water has implication on the population health. Availability of Improved Drinking Water Sources (IDWS) is a problem in developing countries, but variation exists across segments of the population. This study therefore examined the relationship between wealth status, sex of household head and source of potable water.

**Methods:**

The 2013 Nigeria Demographic and Health Survey data was used. A representative sample of 40,680 households was selected for the survey, with a minimum target of 943 completed interviews per state covering the entire population residing in non-institutional dwelling units in the country. Households where information on drinking water sources was not reported were excluded, thus reducing the sample to 38021. The dependent and key independent variables were IDWS and Wealth Index respectively. Data were analysed using Chi-square and binary logistic regression (α = .05).

**Results:**

Households that used IDWS were headed by females (66.7 %) than males (58.7 %). Highest proportion of households who used IDWS was found in the rich wealth index group (76.7 %). The likelihood of using IDWS was higher in household headed by females (OR = 1.41; C.I = 1.33–1.49, *p* <0.001). Households that belong to rich wealth index and middle class were 5.06(C.I = 4.81–5.32, *p* <0.001) and 2.62(C.I = 2.46–2.78, *p* <0.001) respectively times more likely to IDWS than the poor. This pattern was sustained when other confounding variables were introduced into the regression equation as control.

**Conclusions:**

Households headed by women used improved drinking water sources than those headed by men. However, wealth index has strong influence on the strength of relationship between sex of household head and improved drinking water sources.

## Background

Water is one of nature’s most important gifts to mankind. Access to safe drinking water is a basic need for human growth and development. Safe drinking water reduces the chances of contacting waterborne diseases and infections [1]. Water influences health through direct consumption for drinking and for sanitation, and for its use in food and nutrition in the households [1]. While the developed nations have made adequate provisions for safe drinking water for their citizens, the developing nations are just striving to achieve this goal.

Despite failure of Government in developing countries to provide potable water to households, individuals tried to ensure that the source of water they drink in their homes is safe. This at times is done by sinking boreholes and tube wells in their houses. The cost of making such provision is huge and thus making it unbearable to people in a country like Nigeria where majority of its citizens live below the poverty line. In Nigeria, there exists a wide gap between the rich and poor in terms of meeting their daily needs including provision of essential amenities like water. Water, health and poverty are closely linked to each other. Health and poverty have two-way relationship: good health brings prosperity, and prosperity brings improvements in health; or conversely poor health may create and perpetuate poverty and poverty may lead to poor health. Poverty is now recognised as lack of access to different livelihood capitals, such as water [2].

However, the sex of the household head can make a difference in the provision of safe drinking water. In Nigeria context, the culture demands that men should be the head of household except few situations where women may take up such responsibility. Sex of the head of the household plays a role among the determinants of household choice of water source. Evidence has shown that male-headed households are less likely to choose an improved source than do female-headed households [3].

The human right to water entitles everyone to sufficient, safe, acceptable, physically accessible and affordable water for personal and domestic uses [4]. Though essential for human life, more than 1 billion people in the world do not have access to safe drinking water. In developing countries, contaminated drinking water is a major health hazard [4] and water-related diseases are a significant contributor to the global burden of illnesses. The common diseases here are waterborne: 1.8 million people die every year from waterborne diseases like cholera [5]. Furthermore, 21 % of infant mortality in developing countries is caused by diarrhoeal diseases [2]. Moreover, 88 % of the cases of diarrhoeal disease are attributed to unsafe water supply, inadequate sanitation and hygiene [2]. In recent years, access to safe and reliable water supplies has received increased attention around the world. At the United Nations Millennium Summit in 2000 and subsequently at the Johannesburg Earth Summit in 2002, world leaders agreed to a set of time-bound and measurable development targets widely known as the Millennium Development Goals for 2015 which include a commitment to halve the proportion of people without access to safe drinking water [6].

According to the United Nations Development Program [7], nearly one-sixth of the world’s population obtains drinking water from unimproved sources, and in many developing areas, progress in expanding clean water coverage is modest. From a total access of 77 % in 1990, today, the World population’s access to improved water has increased to 87 % [8]. However, in Nigeria, only 58 % access to improved water is currently recorded [8]. This represents a slight growth in access from 1990 when the proportion was 47 % [8].

The household-headship approach in the studies of gender inequalities and poverty resulted from the fact that the household has been the unit of analysis for studying poverty and female headship was the only gender-transparent factor in this approach. Even though female-headed households are a relatively small proportion of households, evidence shows that in the last 20 years, their share in the total is increasing in most regions of the world [9]. The fact that male-headed households are predominantly more financially secured does not mean that they have more access to improved drinking water than female-headed households. This depends on how preferences and priorities between these two households may differ.

Household wealth plays significant role in demand for drinking water quality and access to potable water [10]. Bosch et al. [11] stated that fewer poor households are connected to water networks and many poor households have access to lower-quality services than non-poor households. Micro studies in urban areas globally show that the urban poor are disproportionately underserved in the distribution of public utility, and hence consume poor quality and little water [12]. Being deprived of clean water might be derived from being “income poor” due to lack of clean water. In regions where water supply is adequate and reliable, people’s income may be an important determinant of the source of drinking water [13].

Globally, the aphorism that “water is life” incontestably rings true. But for the large percentage of Nigerians who lack access to potable water, the resource could be said to be a harbinger of diseases, ill-health and, ultimately, death. It should not be so in a country that has vast freshwater resources and huge public revenues to provide quality water for its citizens. On a daily basis, many Nigerians engage in an unending struggle to get water for hydration, sanitation and hygiene. It is really a vicious cycle. Many of them trek several kilometres to get any form of water at all. Although its purity cannot be guaranteed, sachet water – or “Pure Water” in local parlance – has become a way of life for many Nigerians.

The conceptualisation of the study was based on backdrop of limited research evidence on the relationship between gender of household heads, wealth and sources of drinking water in Nigeria. This study was therefore designed to examine the role of poverty in the gender of household heads and the choice of sources of potable water supply in Nigeria.

## Methods

### Study area

The 2006 Population and Housing Census reported Nigeria’s population to be 140,431,790, with a national growth rate estimated at 3.2% per annum. With this population, Nigeria is the most populous nation in Africa, as noted, and the seventh most populous in the world. The three major ethnic groups in Nigeria are; Hausa, Yoruba, and Igbo. Presently, Nigeria is made up of 36 states and a Federal Capital Territory, grouped into six geopolitical zones: North Central, North East, North West, South East, South South and South West. There are 774 constitutionally recognised Local Government Areas (LGAs) in the country. Potable water is a problem in Nigeria with 42 % of the population still gets their drinking water from unimproved sources [14].

### Data collection procedures

The study utilised secondary data (Nigeria Demographic Health and Survey, 2013) obtained from the MEASURE DHS: Demographic and Health Surveys website after the approval for its use was granted by the data originators. Cross-sectional design was used for data collection and the survey covered the entire population residing in non-institutional dwelling units in the country.

The survey used as a sampling frame the list of enumeration areas (EAs) prepared for the 2006 Population Census of the Federal Republic of Nigeria, provided by the National Population Commission. The sample was designed to provide population and health indicator estimates at the national, zonal, and state levels. The primary sampling unit (PSU), referred to as a cluster in the 2013 NDHS, is defined on the basis of EAs from the 2006 EA census frame. The sample was selected using a stratified three-stage cluster design consisting of 904 clusters, 372 in urban areas and 532 in rural areas. A representative sample of 40,680 households was selected for the survey, with a minimum target of 943 completed interviews per state. For detailed information on sample size and design, the interested readers should access the information online at (www.measuredhs.com). In this study, households where information on drinking water sources was not reported were excluded, thus reducing the sample to 38021.

### Variable description

The dependent variable was source of potable water. In the original data, different sources of drinking water were mentioned by the respondents. We therefore Theses sources were categorised into improved and unimproved. Sources that are likely to provide water suitable for drinking are identified as improved sources. These include a piped source within the dwelling, yard, or plot; a public tap/stand pipe or a borehole; a protected well or spring; and rainwater [15].

The independent variables of interest were sex of head of household classified as male or female and wealth quintile which was originally grouped as poorest, poor, middle, richer and richest. However, due to small number of respondents in some of the wealth quintile categories, the variable was re-categorised in this study as poor, middle and rich. Other independent variables used are age of the household head, highest level of education, religion, place of residence, region, time to get to drinking water source and number of household members.

### Methods of analysis

Data were analysed at bivariate and multivariate levels using Chi-square and binary logistic regression. At multivariate level of the analysis, three logistic regression models were generated to examine the factors influencing the use of potable water sources. The first model examined the independent relationship between the sex of the household head, wealth index and drinking water sources while the second model involves the interaction between the two key independent variables (wealth index and drinking water) and the dependent variable (drinking water sources). In the third model, all other dependent variables (socio-economic and health related variables) were included in the regression equation as control.

### Ethical approval

The data originators obtained ethical approval from Nigeria National Ethics Committee (NNEC) functioning under the Ministry of Health. An informed consent was obtained from all the study participants after explaining to them all the issues related to the study in details at the point of data collection. Eligible respondents who did not want to partake in the study were excluded from the survey. Participants that consented to be involved in the study were made to sign appropriate agreement form before the interview. Also an approval to use the data for this study was granted by the data originator before data access and subsequent retrieval.

## Results

About 27.6 % of the study subjects were in the 35–44 age groups. Thirty-eight percent had no formal education while 9.0 % had higher education. Slightly above half, 52.3 % and 57.9 % were Muslims and live in rural areas respectively. About one-third of respondents was from the North western part of Nigeria and had more than seven persons in their household. Majority (83.2 %) of respondents had their homes headed by a male. About 38.0 % and 43.2 % of the women interviewed were in the poorest and richest wealth quintile (Table 1).

**Table 1:** Socio-demographic characteristics of Respondents, Demographic and Health Survey, 2013, Nigeria

Background characteristics	Number	Percent
Total	38021	100.0
Sex of household head		
Male	31631	83.2
Female	6390	16.8
Age of the household head		
15 – 24	1464	3.9
25 – 34	7479	19.7
35 – 44	10505	27.6
45 – 54	9248	24.3
55 – 64	5749	15.1
65+	3576	9.4
Highest educational level		
No formal education	14466	38.1
Primary	6594	17.3
Secondary	13550	35.6
Higher	3411	9.0
Religion		
Christian	17766	46.7
Islam	19893	52.3
Traditional	362	1.0
Place of residence		
Urban	16020	42.1
Rural	22001	57.9
Region		
North Central	5429	14.3
North East	5664	14.9
North West	11704	30.8
South East	4347	11.4
South South	4668	12.3
South West	6209	16.3
Number of household members		
1 – 3	7713	20.3
4 – 5	10080	26.5
6 – 7	8776	23.1
8+	11452	30.1
Wealth index		
Poor	14298	37.6
Middle	7304	19.2
Rich	16419	43.2
Time to drinking water sources		
On premises	8325	21.9
<30 min	19642	51.7
≥30 min	9789	25.7
Don’t know	266	0.7

In Table 2 the data show that more households that used improved drinking water sources were headed by females (66.7 %) than males (58.7 %) (*p* <0.001). Highest proportion of the respondents with higher education (72.7 %) use water from improved sources. The use of improved drinking water sources were significantly associated with; age of respondents, religion, region and number of persons in a household (*p* <0.001). As expected, households in the urban areas (75.7 %) and those in the richest wealth category (76.7 %) use improved sources of drinking water in their households. Differential in the use of improved sources also existed in the time to get to water source. More women (68.3 %) who had water source located within their premises used water from improved sources than others (*p* <0.001).

**Table 2:** Sources of Household drinking water according to background characteristics, Demographic and Health Survey, 2013, Nigeria

Background characteristics	Improved drinking water sources (%)	Total women	*X* ^2^-value	*p*-value
Total	60.1	38021		
Sex of household head^a^			141.6	<0.001
Male	58.7	31631		
Female	66.7	6391		
Age^a^			2001.0	<0.001
15 – 24	50.2	1464		
25 – 34	55.3	7479		
35 – 44	60.0	10505		
45 – 54	61.4	9248		
55 – 64	64.2	5749		
65+	64.0	3576		
*Mean ± σ*	*45.6 ± 13.7*	*44.8 ± 13.7*		*<0.001*
Highest educational level^a^			182.9	<0.001
No education	47.4	14466		
Primary	59.6	6594		
Secondary	70.7	13549		
Higher	72.7	3412		
Religion^a^			277.6	<0.001
Christian	64.0	17766		
Islam	57.0	19894		
Others	35.9	362		
Place of residence^a^			2824.0	<0.001
Urban	75.7	16020		
Rural	48.7	22001		
Region^a^			742.1	<0.001
North Central	55.1	5429		
North East	49.0	5665		
North West	58.0	11703		
South East	68.4	4347		
South South	69.3	4668		
South West	65.8	6210		
Number of household members^a^			48.55	<0.001
1 – 3	60.9	7712		
4 – 5	61.6	10079		
6 – 7	61.1	8777		
8+	57.4	11452		
*Mean ± σ*	*6.4 ± 3.5*	*6.5 ± 3.7*		*0.130*
Wealth index^a^			445.9	<0.001
Poor	39.4	14298		
Middle	63.0	7304		
Rich	76.7	16419		
Time to drinking water source^a^			938.1	<0.001
On premises	68.3	8325		
<30 min	62.9	19641		
≥30 min	48.0	9789		
Don’t know	40.8	265		

^a^Significant at 5.0 %

### Multivariate results

In the first model, the data show that household headed by females used improved water sources (OR = 1.41; C.I = 1.33–1.49, *p* <0.001) than those headed by males. The likelihood of using portable water sources increases consistently with increasing level of wealth index. Households that belong to rich wealth index and middle class were 5.06 (C.I = 4.81–5.32, *p* <0.001) and 2.62 (C.I = 2.46–2.78, *p* <0.001) respectively more likely to use potable water sources than the poor. When wealth index was used solely as the control for the relationship between sex of the household head and potable water sources, the data revealed that the strength of the relationship which was initially significant disappeared (model 2). However, it is striking that significant association existed between sex of household head and potable water sources when other confounding variables were introduced into the regression equation as control. In this case, the likelihood of getting drinking water from improved sources in households headed by females was 1.17 (C.I = 1.09–1.25, *p* <0.001) times higher than that of males (Table 3).

**Table 3:** Multivariate logistic regression of relationship between Household sources of drinking water according to background characteristics, Demographic and Health Survey, 2013, Nigeria

Background characteristics	Model 1		Model 2		Model 3	
	UOR	C.I (UOR)	AOR	C.I (AOR)	AOR	C.I (AOR)
Sex of household head						
Male	1		1		1	
Female	1.41^a^	1.33-1.49	1.02	0.96 – 1.08	1.17^a^	1.09-1.25
Wealth index						
Poor	1		1		1	
Middle	2.62^a^	2.46-2.78	2.61^a^	2.46-2.77	2.69^a^	2.51 – 2.87
Rich	5.06^a^	4.81-5.32	5.05^a^	4.80-5.30	4.49^a^	4.15 – 4.86
Age of household head						
15 – 24					1	
25 – 34					1.15^b^	1.02 – 1.31
35 – 44					1.41^a^	1.24 – 1.60
45 – 54					1.52^a^	1.34 – 1.73
55 – 64					1.73^a^	1.51 – 1.98
65+					1.74^a^	1.51 – 2.00
Highest educational level						
No education					1	
Primary					1.29^a^	1.19 – 1.38
Secondary					1.38^a^	1.28 – 1.49
Higher					1.09	0.98 – 1.22
Religion						
Christian					1	
Islam					1.46^a^	1.36 – 1.56
Others					0.76^b^	0.60 – 0.96
Place of residence						
Urban					1	
Rural					0.56^a^	0.53 – 0.59
Region						
North Central					1	
North East					1.23^a^	1.13 – 1.35
North West					1.65^a^	1.52 – 1.79
South East					1.24^a^	1.12 – 1.37
South South					1.25^a^	1.14 – 1.37
South West					0.63^a^	0.58 – 0.69
Number of household members						
1 – 3					1	
4 – 5					1.03	0.96 – 1.11
6 – 7					1.05	0.97 – 1.13
8+					0.93	0.86 – 1.00
Time to get to drinking water source						
On premises					1	
<30 min					0.95	0.89 – 1.01
≥30 min					0.53^a^	0.49 – 0.57
Don’t know					0.19^a^	0.14 – 0.25
−2 Log likelihood	51011.6		46624.4		44661.2	
Cox & Snell R^2^	0.04		0.112		0.157	

*C.I* confidence interval; *UOR* unadjusted odd ratio; *AOR* adjusted odd ratio

^a^Significant at 0.1 %; ^b^Significant at 5 %

The identified predictors of using improved water sources are; sex of the household head, wealth quintile, age of the household head, highest level of education, religion, place of residence, region and time to get to drinking water source.

## Discussion

Access to improved water, though essential for human life still remains a day to day struggle for hundreds and thousands of citizens who live mainly in developing countries [2, 16, 17]. Household water supply has become an important public policy issue because safe water is an essential component of primary health care. Access to and use of safe drinking water can make an immense contribution to health, productivity, and social development of individuals at micro level and the nation at large [15, 18, 19].

However, many people in developing countries continue to rely on unimproved water sources mainly because of lack of access to potable water [4]. As evidenced in the current study, about two-fifth of households still use water from unimproved sources as drinking water. Lawrence et al. [14] noted that socio-economic status is a significant determinant of household access to water. Other variables closely connected with the availability of water include, among others, gender of the household head and household size [20].

According to Abebaw et al. [3] gender of the head of the household plays a role among the determinants of household choice of water source. Male-headed households are less likely to use water from an improved source than do female-headed households [3]. Our finding corroborates the significance of household heads in the choice of source of drinking water. In this study, we found that the likelihood of getting drinking water from improved sources in households headed by females was higher than that of males. However, this finding is at variance with a study conducted on awareness and the demand for improved drinking water source in Cameroon which showed that male-headed households are less likely to choose an improved source than do female-headed households [21].

Poverty is a multidimensional phenomenon [22]. Our finding also gives credence to the differentials in wealth index in the choice of water from improved sources. The likelihood of using potable water sources increases consistently with increasing level of wealth index. Household wealth index has a statistically significant role in demand for drinking water quality: better-off households are more likely to consume safe and reliable water. This result is consistent with Totouom and Fondo [10] who use the per capita expenditure used as proxy for household welfare and concluded that as households become better-off, they are much more likely to choose improved quality water. It may be argued that the income of individuals from the middle and poorest wealth index may be insufficient in affording the cost of using clean water, which would compel them to use inadequate and unreliable sources of water supply.

This study further revealed that the likelihood of using water from improved sources increased with increasing level of education. That is, the probability for a given household to adopt an improved water source increases with the educational level. The significant effect of education on households’ choice of drinking water source is in conformity with previous studies [3, 23]. Studies on the determinants of households choice of water source in developing countries have proved that household education have a strong influence on households choice [24–26]. This is not surprising, since more educated households are probably more aware about adverse health effects from ingestion of poor water quality.

The number of household members is one of the basic demographic characteristics of a household. The number of people in a household determines whether this household obtains its water from an improved source [21]. Findings from this study reported the likelihood of sourcing water from improved sources increases with increasing number of persons in a household. Although, not statistically significant, the finding might be due to the fact that large households might have sufficient members that share household daily responsibilities including access to improved water at distances far away from home. This is in agreement with findings by de Sherbiniin et al. [27] where it was reported that increase in household size is not associated with obtaining water from an unsafe source. However, this is in contrast to a study conducted in Cameroon where it was reported that the increasing of the size of a household decreases the likelihood of using improved sources [21]. The author argued that households with more members are more likely to be faced with poverty than households with fewer members and so may not be able to access or pay for water from improved sources [28].

Place of residence is a strong determinant of households’ choice to use an improved source. According to the World Bank, large numbers of those who lack access to improved water supply infrastructure live in urban areas but the proportion relative to those in rural areas is low [29]. Living in urban area increases the probability of adopting an improved source. Our study also confirmed that women who live in rural areas are less likely to use water from improved sources than their counterparts in urban areas. In Nigeria, Government focuses more on developmental programmes and social amenities in urban areas than rural [30].

It is evidenced in this study that the use of water from improved sources was more prominent among women who resides in the South South (69.3 %) geopolitical zones of Nigeria than those from the North East (49.0 %). Our findings differ from the survey conducted by the National Bureau of Statistics (NBS) in [31] which reported that improved water coverage ranged from 73.5 to 30.7 %, with the South West Zone having the highest coverage of improved water source and the North East Zone has the lowest coverage. The management of water resources is reported to be ineffective in Nigeria. This is because there is inadequate and inequitable distribution of adequate surface and groundwater supplies; hence there are significant zonal and state variations in the proportion of people using improved water sources [32].

As expected, our study revealed that the odds of using water from improved sources reduce with increase in the distance covered in accessing improved water source. This means that women that trekked for 30 min or more are less likely to use water from improved sources than those with water source sited in their premise. Thus, the longer the distance to a particular source of drinking water, the lower will be the demand for same [33]. It is estimated that women in many developing countries walk for an average of about 6 km each day to collect water [34]. The human right to water entitles everyone to sufficient, safe, acceptable, physically accessible and affordable water for personal and domestic uses. Water near the home produces significant improvements in nutrition and health. The carrying of water over long distances is a health hazard, especially during development and pregnancy periods.

## Conclusions

The gender and wealth of a household head play a significant role in the choice of water source. Other identified factors that predict the choice of water source includes age of the household head, highest level of education, religion, place of residence, region and time to get to drinking water source. Given the growing emphasis being placed on population access to improved water sources in achieving the Millennium Development Goals (MDGs), there is the need for policy makers and relevant stakeholders to more accurately target initiatives for population access to potable water. Some common tropical diseases are water-related and can be eliminated if adequate supply of water at the right quantity and quality is provided to the public. People should be equipped with improved water sources as close as possible; otherwise, they will rely on nearest unimproved sources. Reducing inequalities as regarding wealth and investing in education and the water sectors would play a key role in having a country in which her citizens have access to safe, affordable and reliable improved water sources.

## Abbreviations

AOR: Adjusted Odd Ratio
C.I: Confidence Interval
EAs: Enumeration areas
IDWS: Improved Drinking Water Sources
LGAs: Local Government Areas
MDGs: Millennium Development Goals
NBS: National Bureau of Statistics
NDHS: Nigeria Demographic Health and Survey
NNEC: Nigeria National Ethics Committee
PSU: Primary Sampling Unit
UOR: Unadjusted Odd Ratio
